# Emergence of Difficult-to-Treat Tinea Corporis Caused by *Trichophyton mentagrophytes* Complex Isolates, Paris, France

**DOI:** 10.3201/eid2801.210810

**Published:** 2022-01

**Authors:** Sarah Dellière, Brune Joannard, Mazouz Benderdouche, Anselme Mingui, Maud Gits-Muselli, Samia Hamane, Alexandre Alanio, Antoine Petit, Germaine Gabison, Martine Bagot, Stéphane Bretagne

**Affiliations:** Hôpital Saint Louis Laboratoire de Parasitologie-Mycologie, Assistance Publique des Hôpitaux de Paris, Paris, France (S. Dellière, B. Joannard, M. Benderdouche, A. Mingui, M. Gits-Muselli, S. Hamane, A. Alanio, S. Bretagne);; Université de Paris, Paris (S. Dellière, M. Gits-Muselli, A. Alanio, M. Bagot, S. Bretagne);; Centre National de Référence Mycoses Invasives et Antifongiques Unité de Mycologie Moléculaire, Paris (A. Alanio, S. Bretagne);; Hôpital Saint-Louis Service de Dermatologie, Assistance Publique des Hôpitaux de Paris, Paris (A. Petit, G. Gabison, M. Bagot); INSERM U976 (M. Bagot)

**Keywords:** antimicrobial resistance, *Trichophyton mentagrophytes* complex, *Trichophyton indotineae*, squalene epoxidase, terbinafine, skin infections, antifungal drug resistance, fungi, human-to-human transmission, France

## Abstract

We describe 7 cases of extensive tinea corporis since 2018 in a hospital in Paris, France, after failure to cure with terbinafine. Molecular analysis indicated *Trichophyton mentagrophytes* internal transcribed spacer type VIII (*T. indotineae*). This strain, which has mutations in the squalene epoxidase gene, is spreading on the Indian subcontinent.

Dermatophytes are filamentous keratinophilic fungi responsible for superficial fungal diseases involving the skin, hair, or nails. On the basis of the site of the lesions, conditions are called tinea corporis, tinea cruris, tinea capitis, tinea pedis, or tinea unguium. Since the mid-2010s, difficult-to-treat cases of tinea corporis and tinea cruris have emerged in India; molecular analysis revealed that this clinical presentation was caused by a unique clade related to the *Trichophyton mentagrophytes* complex (*1*). Since 2018, we have observed several cases of clinically resistant tinea corporis with extensive lesions that do not respond to terbinafine, the preferred first-line treatment. We investigated the microbiological origin of this resistance by looking for mutations in the squalene epoxidase (SQLE) gene, which terbinafine targets, and determining the MICs of antifungal drugs.

## The Study 

**Figure 1:**
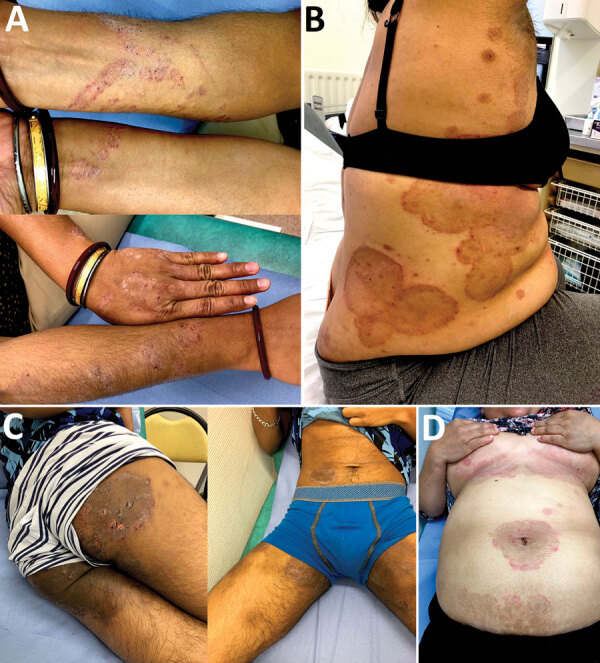
Morphologic features of difficult-to-treat dermatophytosis caused by *Trichophyton mentagrophytes* complex internal transcribed spacer type VIII (*T. indotineae*) in patients in Paris, France. A) Scaly plaques with erythema and surrounding papulae and vesicles of the arms (patient 1); B) centrifuge annular erythema of the trunk after topical and oral corticosteroids (patient 6); C) erythematous and scaly plaques (patient 3); D) pruritic cutaneous lesions of the groin and axillary pits to which was applied steroids (patient 5).

During January 1, 2018–December 31, 2019, we saw 2,282 patients for dermatophytosis at Hôpital Saint-Louis Parasitologie-Mycologie (Paris, France). Of these, 350 (15.3%) patients were positive for *T. mentagrophytes* complex, identified by macroscopic and microscopic phenotypical features. Seven (2.0%) patients, all of whom were either recent immigrants or born in a country on the Indian subcontinent and had traveled back to their birthplace in recent years, had clinically terbinafine-resistant tinea corporis (Table 1). The cutaneous lesions were often multiple and extensive and involved the groin, axillary pits, trunk, limbs, and face but spared the extremities and nails (Figure 1). The patients’ main complaint was intractable pruritus. 

**Table 1:** Clinical characteristics and treatment for patients with difficult-to-treat tinea corporis caused by *Trichophyton mentagrophytes* complex isolates, Paris, France*

Patient no.	Age, y/sex	Geographic origin	Medical history	Clinical presentation	First-line treatment	Second-line treatment	Third-line treatment
1	28/F	Bangladesh	None	Erythematous scaly plaques of trunk and arms; pruritus (Figure 1, panel A)	TBR 250 mg/d; outcome (9 wk): partial healing, positive culture of skin sample	GSF 500 mg x 2/d; outcome (4 wk): no healing	ITZ 200 mg/d; outcome (12 wk): improvement, negative culture of skin sample; relapse 5 mo after ending ITZ
2	47/F	India	Diabetes mellitus, psoriasis	Erythematous scaly plaques of groins and axillary pits; pruritus	TBR 250 mg/d; outcome: no improvement, positive culture of skin sample	ITZ 200 mg/d; outcome (12 wk): healing and negative culture of skin sample; relapse 1 y later	NA
3†	20/M	India	None	Erythematous scaly plaques of groins trunk, buttocks, and legs; pruritus (Figure 1, panel B)	TBR 250 mg/d; outcome (12 wk): no improvement, positive culture of skin sample	ITZ 200 mg/d; outcome (8 wk): healing and negative culture of skin sample	NA
4	46/M	Bangladesh	Diabetes mellitus, dyslipidemia	Plaques with strong pruritic erythema and vesicles with surrounding papulae of groins, buttock, thigh, arms, and face‡	TBR 250 mg/d; outcome (8 wk): no improvement, positive culture of skin sample	ITZ 200 mg/d + topical bifonazole; outcome (12 wk): complete healing	NA
5	44/F	Bangladesh	Diabetes mellitus, dyslipidemia	Erythematous scaly plaques of groins and axillary pits with secondary extension to trunk and limbs‡ (Figure 1 panel D)	TBR 250 mg/d + topical ciclopirox; lost-to-follow up	NA	NA
6	39/F	India	Chronic hepatitis B	Centrifuge annular erythema of trunk and arms;‡ pruritus (Figure 1 panel C)	FCZ 200 mg/wk + topical TBR; outcome (16 wk): improvement; relapse 1 y later	NA	NA
7	57/M	Sri Lanka	Crohn’s disease, psoriasis	Erythematous scaly plaques of groins, buttocks, knees, shoulders, and neck	Topical bifonazole; outcome (8 wk): no improvement	TBR 250 mg/d + topical steroids (for severe associated psoriasis lesions); outcome (8 wk): partial improvement	TBR 250 mg/d; outcome (8 wk): improvement; relapse 1 year later

*FCZ, fluconazole; GSF, griseofulvine; ITZ, itraconazole; NA, not applicable; TBR, terbinafine. †Patient 2’s son. ‡Had applied topical steroids.

We analyzed the 7 clinically resistant *T. mentagrophytes* complex isolates and 8 control isolates from patients with terbinafine-susceptible clinical presentations of tinea (6 cases of tinea pedis or tinea unguium and 1 each of tinea faciei and tinea corporis) collected September 1–October 15, 2018. We sequenced the internal transcribed spacer (ITS) using ITS1 and ITS4 primers, which allowed us to ascribe the isolates to 1 of the 7 different genotypes of *T. mentagrophytes* or the 2 genotypes of *T. interdigitale* (*2*,*3*). We aligned sequences using Geneious Prime version 2020.0.4 software (https://www.geneious.com) and compared them using the Mycobank DNA database (https://www.mycobank.org).

We amplified the SQLE gene of all studied isolates using previously reported primer pairs (*4*). To ease the SQLE gene sequence analysis, we shortened the PCR products by designing 2 internal primers, SQLE-F2 (5′-[658]-TGGGGCCTGGAGCTTATAGA-[677]-3′) and SQLE-R2 (5′-[885]-CCTTCTCCAACGCAGCTTCA-[904]-3′). We compared our sequences to wild-type *T. mentagrophytes* complex reference sequence EZF33561 from GenBank (*5*) and submitted the new sequences to GenBank (accession nos. MW898018–32). We determined MICs as recommended (*6*). We obtained approval from the Commission Nationale de l’Informatique et des Libertés, the national data-protection agency in France (approval no. 903395), to ensure that patient data would be kept anonymous according to national regulations.

**Figure 2:**
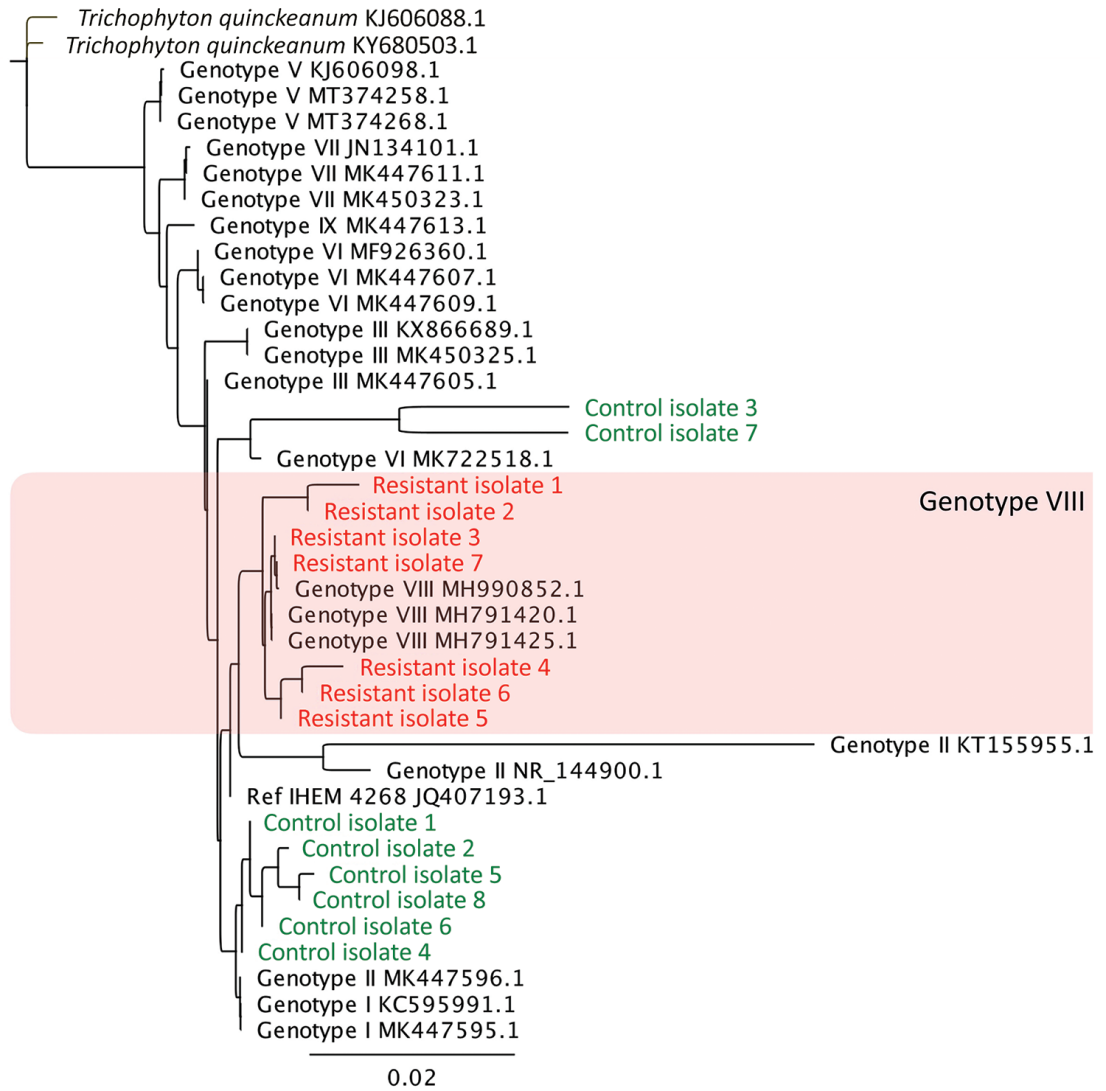
Phylogenetic tree of clinically resistant *Trichophyton mentagrophytes* complex internal transcribed spacer (ITS) type VIII (*T. indotineae*) isolates from patients in Paris, France (red) and control isolates (green) compared with reference isolates. Isolates were classified on the basis of the ITS regions of rDNA classified among existing isolates from type I to type IX strains retrieved from GenBank (*3*). All resistant isolates and control isolates 2 and 8 had non–wild-type SQLE sequence and different MIC profiles (Table 2). Tree was constructed based on maximum-likelihood (Tamura-Nei model) analysis including sequences of the reference *Trichophyton mentagrophytes* neotype strain (IHEM 4268^NT^). Reference isolates are identified by GenBank accession number; *T. quinckeanum* (Genbank accession nos. KJ606088.1 and KY680503.1) was used as outgroup.

We generated a phylogenetic tree based on alignment of the ITS sequences (Figure 2). The 7 resistant isolates (Table 2) belonged to *T. mentagrophytes* ITS type VIII, whereas 6/8 control isolates belonged to a different subgroup close to genotypes I and II associated with the anthropophilic *T. interdigitale* and 2/8 to genotype VI (*3*). SQLE sequencing revealed substitutions in the 7 resistant isolates at sequences F397L, L393S, and A448T (Table 2). We also observed polymorphisms (at K276N) in 2 of the 8 control isolates (Table 2). We determined MICs of antifungal drugs for the 9 isolates with non–wild-type SQLE sequences (Table 2). These MICs were not homogenous, according to the SQLE sequences. The 2 isolates with the A448T substitution had low terbinafine MICs, similar to the 2 control isolates with the K276N substitution (Table 2). Only 1 isolate (from patient 7) with the A448T substitution had a high MIC for azoles.

**Table 2:** MICs of the 9 isolates with non–wild-type SQLE sequences from patients with difficult-to-treat tinea corporis caused by *Trichophyton mentagrophytes* complex isolates, Paris, France, and control isolates*

Drug	F397L		L393S		A448T		K276N

Patient 1	Patient 2	Patient 3	Patient 4	Patient 5	Patient 6	Patient 7	Control 5	Control 8
Terbinafine	4	4	4	4		1		0.014	0.03		0.03	0.03
Itraconazole	0.014	0.06	0.06	0.014		0.06		0.06	16		0.25	0.25
Voriconazole	0.25	0.125	0.25	0.25		0.25		0.25	2		0.25	0.25
Posaconazole	0.03	0.06	0.06	0.03		0.06		0.06	0.5		0.25	0.125
Isavuconazole	0.125	0.25	0.5	0.125		0.5		0.25	4		0.5	0.5

*SQLE, squalene epoxidase.

## Conclusions

We describe a series of cases of tinea corporis in France caused by *T. mentagrophytes* complex ITS type VIII (*3*), which showed sufficient molecular and phenotypic differences to be recently individualized into a separate species called *T. indotineae* (*7*). Although its frequency is currently low (2.0% of all *T. mentagrophytes* complex isolates in our hospital), it is alarming because of the therapeutic failure we observed when using available antifungal drugs (*8*). *T. indotineae* is endemic to India (*1*) and Iran (*2*,*9*), but several cases have been reported in Germany (*3*), Denmark (*6*), Poland (*10*), Belgium (*11*), and Switzerland (*12*). These cases are mainly among persons, similar to our patients, returning from the Indian subcontinent. Direct human-to-human transmission is probable between family members in our study, as reported elsewhere (*9*) and in a couple reported in Switzerland (*12*). However, the possibility that the patients were contaminated from a common source cannot be excluded. Until now, 2 German-born residents have been reported infected despite not having traveled, generating fear of possible extension beyond the initial endemic focus area (*3*).

Thus far, 10 missense mutations in the SQLE gene have been previously proven in vitro to lead to elevated MICs for terbinafine by genetic manipulation (*4*,*13*). The F397L and L393S mutations observed in 5 of our patients have been frequently reported in India and Iran (*1*,*2*), as well as in Europe among travelers or migrants (*3*,*4*,*8*,*10*). A448T substitution was observed in 2 patients, for which both isolates had low terbinafine MICs, and 1 of them had high MICs for azoles, as reported elsewhere (*3*). With the generalization of sequencing, probability of identifying polymorphisms, such as the K276N substitution, increases (Table 2). These observations could be misleading if observed in clinically resistant isolates without performing formal genetic experiments. Moreover, mechanisms other than SQLE mutations have been reported that possibly explain in vitro resistance (*14*). As a consequence, identifying *T. indotineae* seems more clinically relevant than identifying the polymorphism in the SQLE sequence to predicting the failure of antifungal drugs. Without being able to identify the original reservoir of this dermatophyte, one suggestion is that mutations in SQLE and high MICs could be consequences of multiple previous treatments. Delays in seeking specialized medical advice for chronic cutaneous lesions are common, so improper use of topical steroids or over-the-counter medications for alternative diagnoses could favor the accumulation of mutations in the microorganism.

Once *T. indotineae* is identified, the challenge of curing the cutaneous lesions remains. After terbinafine failure, in the absence of contraindications, patients in our study were mainly treated with itraconazole, as recommended (*3*). The long-term success rate of itraconazole was very modest, even when an initial improvement was noted. When checked months after the end of azole treatment, at least 4 of the 7 patients still harbored clinical lesions, and the failure to cure was mycologically confirmed. Of course, every cofactor favoring dermatomycosis should be controlled or avoided when possible, including diabetes (2/7 patients in our study) or use of topical steroids (4/7 patients).

Our findings provide additional evidence of the spread of some dermatophyte species through travel and immigration, as has been evidenced by previously nonendemic *T. tonsurans* replacing other species as the etiologic agent of tinea capitis in children in the Paris area (*15*). Surveillance should focus more specifically on identifying *T. indotineae* than SQLE sequences or MIC testing. Optimal treatment when terbinafine resistance is demonstrated, given the high failure rate of itraconazole, remains to be established.
